# Crop diversification and livelihoods of smallholder farmers in Zimbabwe: adaptive management for environmental change

**DOI:** 10.1186/s40064-016-2802-4

**Published:** 2016-07-19

**Authors:** Clifton Makate, Rongchang Wang, Marshall Makate, Nelson Mango

**Affiliations:** UNEP-Tongji Institute of Environment for Sustainable Development, Tongji University, Shanghai, 200092 China; Key Laboratory of Yangtze Aquatic Environment (MOE), State Key Laboratory of Pollution Control and Resource Reuse, College of Environmental Science and Engineering, Tongji University, Shanghai, 200092 China; University at Albany, State University of New York, 1400 Washington Ave, Albany, NY 12222 USA; International Centre for Tropical Agriculture (CIAT), Kampala, Uganda

**Keywords:** Climate smart agriculture, Crop diversification, Livelihoods, Conditional mixed process, Smallholder farmers, Zimbabwe

## Abstract

This paper demonstrates how crop diversification impacts on two outcomes of climate smart agriculture; increased productivity (legume and cereal crop productivity) and enhanced resilience (household income, food security, and nutrition) in rural Zimbabwe. Using data from over 500 smallholder farmers, we jointly estimate crop diversification and each of the outcome variables within a conditional (recursive) mixed process framework that corrects for selectivity bias arising due to the voluntary nature of crop diversification. We find that crop diversification depends on the land size, farming experience, asset wealth, location, access to agricultural extension services, information on output prices, low transportation costs and general information access. Our results also indicate that an increase in the rate of adoption improves crop productivity, income, food security and nutrition at household level. Overall, our results are indicative of the importance of crop diversification as a viable climate smart agriculture practice that significantly enhances crop productivity and consequently resilience in rural smallholder farming systems. We, therefore, recommend wider adoption of diversified cropping systems notably those currently less diversified for greater adaptation to the ever-changing climate.

## Background

Recent evidence suggests that climate change is emerging as one of the major threats to development across the African continent (Nyasimi et al. 2014). Agriculture is one of the sectors significantly affected by climate change and variability. Seasonality dynamics, increased frequency of droughts (especially mid-season dry spells), increased temperatures, and altered patterns of precipitation and intensity are some of the extreme weather events evident in southern Africa. Declining crop yields, increased agricultural risks, diminishing soil fertility and environmental degradation are some of the main challenges which continue to threaten societal goals of improving food, income and nutrition security especially in smallholder farming. It, therefore, calls for a significant transformation in African agriculture especially in worst affected regions like southern Africa to withstand the emerging challenges. An acceptable and meaningful transformation will be expected to improve productivity, build resilience to farming systems, improve livelihoods and reduce harm to the environment (Nyasimi et al. 2014).

However, climate change adaptation research in agriculture has identified climate-smart agriculture (CSA) as one of the many sustainable agricultural practices (SAPs) that can make households withstand the deleterious effects of climate change and variability in smallholder farming systems (Manda et al. 2016). Hundreds of technologies, practices and approaches fall under the heading of CSA. Such critical practices and techniques include; crop diversification through rotations and intercropping, agroforestry, conservation tillage, cultivation of drought-resistant crops, water harvesting, and integrated soil fertility management, among others (Faurès et al. 2013). CSA is an integrated approach to the implementation of agricultural development programming policies that endeavors to improve productivity, livelihood and environmental outcomes (Rosenstock et al. 2016). Experts, policymakers and other stakeholders concerned about the impact of adverse externalities generated by climate change on the welfare, food, and nutrition security have largely recommended CSA adaptation as an essential vehicle to better the livelihoods of vulnerable segments of the population. CSA is the commonly preferred method to deal with the deleterious consequences of climate change and variability in the smallholder farming sector (FAO 2010; WorldBank 2014). Moreover, it is considered sustainable and a certain practice as it strengthens resilience in smallholder agricultural systems. Its adoption as an adaptation strategy is expected to help smallholder farmers adapt to climate change and variability by intensifying and or diversifying their livelihood strategies.

CSA is premised on three main principles. These include (1) addressing climate-related risk while improving food, income and or nutrition security; (2) achieving productivity and livelihood benefits; (3) having area-specific technologies that suit the specific areas in which they are practiced (Rosenstock et al. 2016). Where possible, CSA also aims to reduce greenhouse gas (GHG) emissions mainly through enhancing the carbon sink (vegetative cover) (Lipper et al. 2014).

The important question we seek to ask is whether CSA technologies at the farm level significantly contribute to crop resilience (adaptive capacity), climate change mitigation and productivity? Despite the theoretical benefits associated with CSA techniques often discussed in the literature, no study to date has attempted to examine the impact of its adoption on productivity and livelihoods in southern Africa, Zimbabwe in particular. This paper contributes to the scarce literature by providing new evidence on the impact of CSA adoption on farm productivity and livelihood outcomes. Particularly, we focus on the impact of crop diversification on smallholder farm productivity and livelihood outcomes in eastern and central Zimbabwe. While crop diversification is not a new practice, the birth of a new challenge “climate change” in agriculture has made it attain popularity as embracing it may significantly reduce risks associated with agricultural production, improve productivity, food security, income and nutrition in smallholder farming systems. The following sub-section outlines the reasons why crop diversification is considered a climate smart technique.

### Crop diversification as a CSA practice

Crop diversification is the practice of cultivating more than one variety of crops belonging to the same or different species in a given area in the form of rotations and or intercropping. It is perceived as one of the most ecologically feasible, cost effective, and rational ways of reducing uncertainties in agriculture especially among smallholder farmers (Joshi 2005). Also, crop diversification increases resilience and brings higher spatial and temporal biodiversity on the farm (Holling 1973; Joshi 2005). According to Lin (2011), crop diversification improves soil fertility, controls for pests and diseases, and brings about yield stability, nutrition diversity, and health. It can also serve as a superior substitute for the use of chemicals to maintain soil fertility and control pests. Truscott et al. (2009) considers crop diversification an environmentally sound alternative to the control of parasites and in the maintenance of soil fertility in agriculture. Diversified cropping systems, in general, tend to be more agronomically stable and resilient. This resilience is mainly because they are usually associated with reduced weed and insect pressures, reduced need for nitrogen fertilizers (especially if the crop mix include leguminous crops), reduced erosion (because of cover crops inclusion), increased soil fertility and increased yield per unit area (Lin 2011). Moreover, diversified cropping systems can also provide habitats for beneficial insects, and this can help reduce the number of pests by rendering host crops less apparent for colonization by parasites. Shoffner and Tooker (2012) attributes the increasing popularity of crop diversification owing to its support for species mixtures over monoculture which offers reasonable ways of controlling pests and diseases. Crop mixtures are mostly like to work by increasing natural enemies of insect pests, breaking the disease cycles, suppressing weeds and volunteer crop plants thereby creating a dilution effect by reducing resource concentration and modification of the microenvironment within the crop canopy and or making pest and diseases pathogen penetration more difficult. Also, crop diversification can contribute to local biodiversity especially when farmers grow indigenous crop varieties. Soil fertility improvement as one of the benefits of crop diversification is a foundation of sustainable and productive farming systems (Lin 2011). Well-managed soils help lower pest pressure, optimize water use by plants, and improve overall crop yields. Moreover, there is also some opinion that crop diversification has a positive impact to climate change effects through the ability of local flora (as opposed to monoculture) to hold carbon thus generating less carbon dioxide. It therefore implies that crop diversification contributes in one way or the other to all the three main principles of CSA by improving; productivity, livelihood outcomes, resilience of farming systems and reducing carbon dioxide emissions. In this paper, we consider crop production activities both in summer and winter growing seasons hence incorporating both crop rotations (e.g. Maize grown in summer and beans planted soon after harvesting maize) and intercropping (e.g. maize-legume intercrops) as part of crop diversification. This is the basis we consider crop diversification as a CSA practice in this paper.

## Research methodology

### Empirical model

For us to examine the impact of crop diversification on farm productivity and livelihoods of smallholder farmers in some parts of rural Zimbabwe, we estimate the following equation with the smallholder farmer as the unit of analysis:

1 $$Y_{i} = \beta_{0} + \beta_{1} \times CD_{i} + \beta_{2} X_{i} + \eta_{1} + \upsilon_{i}$$

where *Y*_*i*_ is a measure of farm productivity or welfare of smallholder farmer *i*; *CD*_*i*_ is a binary variable taking 1 if smallholder farmer *i* practices crop diversification and 0 otherwise; *Xi* is a vector of household or farm level characteristics; *η*_1_ is a term capturing unobserved heterogeneity assumed to be unrelated to the explanatory variables vector *X*_*i*_ and applying to each smallholder farmer living in the same locality; and *υ*_*i*_ captures all the remaining variation with $\upsilon_{i} {\sim}IIDN\left( {0,1} \right)$.

If the vector *X*_*i*_ comprises of all the factors assumed to affect crop diversification including location fixed effects and are uncorrelated with the error *υ*_*i*_, then an ordinary least squares (OLS) regression of Eq. (1) will yield consistent estimates. In that case, our coefficient of interest *β*_1_, which measures the effect of the extent of crop diversification can thus be regarded as the true impact of crop diversification on smallholder farm productivity and welfare.

However, the decision by a smallholder farmer to diversify their crops is potentially an endogenous variable, and failure to control for this endogeneity may result in inconsistent estimates. The endogeneity bias of crop diversifications arises due to the voluntary nature of crop diversification. For example, some smallholder farmers might opt to practice crop diversification simply because they possess more knowledge about the benefits of such practices compared to their counterparts. This type of selection bias will overstate the actual impact of crop diversification in a regression model of the kind specified in Eq. (1). On the other hand, ill-advised smallholder farmers might fail to adopt crop diversification practices merely because they have an informational disadvantage regarding its benefits. In this instance, a failure to control for this kind of bias underestimates the supposed true benefit of crop diversification. Crop diversification (CD), a potentially endogenous variable, takes the following form:

2 $$CD_{i}^{*} = \alpha_{0} + \alpha_{1} {\rm Z}_{i} + \alpha_{2} X_{i} + \eta_{2} + \varepsilon_{i}$$

where $CD_{i}^{*}$ is the propensity to practice crop diversification. However, $CD_{i}^{*}$ is unobserved and what we observe instead is the following:

$$CD = \left\{ {\begin{array}{*{20}l} 1 \hfill &\quad {if\, crop\,\,diversification\; score > 0} \hfill \\ 0 \hfill &\quad {otherwise} \hfill \\ \end{array} } \right.$$

In this case, we created a crop diversification index^1^ measuring the extent of crop diversification for each smallholder farmer which we then use to create the binary variable *CD* equals 1 if the crop diversification score is greater than 0 (implying some diversification by smallholder farmer) and 0 otherwise. The vector Z_*i*_ contains a set of variables thought to influence crop diversification such as management and technical abilities of smallholder farmers and acquired knowledge through contact with agricultural extension workers (Abdulai and Huffman 2014; Manda et al. 2016); *η*_2_ is the unobserved heterogeneity parameter assumed to be uncorrelated with the vector of explanatory variables (*X*_*i*_) and *ε*_*i*_ captures the remaining unobserved variation. The subscripts {1, 2} in the unobserved heterogeneity components (*η*) are equation indicators.

The standard approach in the economics literature to control for endogeneity bias is to estimate Eq. (1) with instrumental variables for crop diversification [Eq. (2)]. Instrumental variables are those variables highly correlated with the endogenous variable (crop diversification in this case) and not correlated with the unobserved factors that may affect the outcome variables (Angrist et al. 1996; Angrist and Krueger 2001). However, as is well known, it is very difficult to obtain good instruments. To avoid the problems often associated with poor instruments, we jointly estimate Eqs. (1) and (2).

### Joint estimation

As mentioned earlier, the endogeneity nature of crop diversification can significantly over or under-estimate the impact of crop diversification on farm productivity and welfare. To control for this possibility, we jointly estimate Eqs. (1) and (2) within a Conditional (recursive) Mixed Process (CMP) framework introduced by Roodman (2011). The CMP controls for the selection bias that arises from unobserved factors affecting our outcome variables by building from the seemingly unrelated regression framework and allowing for cross-equation correlation of the error terms. Allowing for the potential endogeneity of crop diversification in Eq. (1), we can express the joint marginal likelihood as follows:

3 $$\int\limits_{{\eta_{2} }} {\int\limits_{{\eta_{1} }} {\left[ {\prod L_{2} \left( {\eta_{2} } \right)\prod L_{1} \left( {\eta_{1} } \right)} \right]} } f\left( {\eta_{2} ,\eta_{1} } \right)d\eta_{2} d\eta_{1}$$

where *L*_1_ and *L*_2_ are the conditional likelihood functions of Eqs. (1) and (2) respectively; $f\left( {\eta_{2} ,\eta_{1} } \right)$ is the joint distribution of the unobserved heterogeneity components. In this case, the joint distribution of the unobserved effects $f\left( {\eta_{2} ,\eta_{1} } \right)$ is assumed to be a two-dimensional normal distribution characterized as follows:

4 $$\left( \begin{aligned} \eta_{2} \hfill \\ \eta_{1} \hfill \\ \end{aligned} \right){\sim}N\left( {\left[ \begin{aligned} 0 \hfill \\ 0 \hfill \\ \end{aligned} \right],\left[ {\begin{array}{*{20}l} {\sigma_{2}^{2} } \hfill & {} \hfill \\ {\rho_{12} \sigma_{2} \sigma_{1} } \hfill & {\sigma_{1}^{2} } \hfill \\ \end{array} } \right]} \right)$$

The complete specification or full model is jointly estimated via the conditional mixed process, CMP which utilizes the Geweke, Hajivassiliou, and Keane (GHK) algorithm to consistently estimate the likelihood function given in (3). As mentioned earlier, the main reason for jointly estimating Eqs. (1) and (2) is to control for potential self-selection bias. As explained in Maitra (2004), joint estimation implies the possibility of non-zero covariance between the error terms of the two Eqs. (1) and (2), i.e. $Cov\left( {\eta_{2} ,\eta_{1} } \right) \ne 0$. However, since we condition on the heterogeneity terms, Eqs. (1) and (2) become independent, making it easy to get the likelihood function in (3) above by simply multiplying the individual conditional likelihood functions of Eqs. (1) and (2) (Chamberlain et al. 1975). Since finding appropriate instrumental variables is a huge challenge, the joint model (with correlated errors) allows us to derive selection-bias revised estimates for smallholder farm productivity, household income and food security as long as the Eqs. (1) and (2) are identified.

### Identification

In this case, identification is ensured by the recursive nature and the covariance restrictions imposed by the addition of a fixed effect in each of Eqs. (1) and (2). In this instance, a recursive structure is ensured by the fact that either of our outcome variables measuring farm productivity, food security and household crop income [Eq. (1)] depends on crop diversification [Eq. (2)] but not vice versa. For a more completed discussion on this issue, the reader is referred to Chamberlain et al. (1975). Following the arguments in Chamberlain et al. (1975), we do not necessarily require a set of instruments to identify the system of equations. Nevertheless, it is generally considered a good practice to include some instrumental variables for the identification of the crop diversification equation. In this paper, we follow related studies examining the impacts of sustainable agricultural practices on agricultural productivity and incomes and use as our identifying restrictions (${\rm Z}_{i}$), variables measuring management and technical abilities of smallholder farmers and acquired knowledge through contact with agricultural extension workers (Abdulai and Huffman 2014; Manda et al. 2016). In particular, we use a dummy variables equals 1 if smallholder farmer has at least one contact with an agriculture extension officer; has access to information regarding output prices; possesses knowledge about the road network in the area; is well-informed about transport costs; has access to any other market intelligence and zero otherwise. We believe that these variables are more likely to have a direct impact on crop diversification and the only channel they can influence productivity, income and food security is through their impact first on crop diversification and not vice versa.

To check the robustness and validity of our empirical findings, we also estimated Eq. (1) using an instrumental variable approach. In this case, we only utilized the dummy variable measuring smallholder farmers’ contact with at least one agricultural extension officer as an instrumental variable for crop diversification (results shown in Table 4). Even though we come to the same conclusions regarding the impact of crop diversification on farm productivity, income, and food security, we interpret these results with caution as we suspect that the problem of weak instruments may arise as indicated by relatively small first stage F-statistics especially for productivity as measured by cereal output. Staiger and Stock (1997) argue that the problem of weak instruments may cause serious bias on the results if the F-test for joint significance does not exceed 10 (see Table 4). We thus focus much on the results of the joint estimation approach. We conduct all our analysis using Stata version 13.

### Data description and sampling

The data utilized in this paper comes from surveys of smallholder farming communities in Zimbabwe’ four districts of Guruve, Wedza, Mudzi and Goromonzi. More than 600 smallholder farmers in the four districts were interviewed. Commissioned by the International Centre for Tropical Agriculture (CIAT), the surveys collected data on a number of characteristics including crop production and management, household composition, household market participation, access to infrastructure, household incomes, ownership of land and non-land assets, livestock ownership,^2^ and access to agricultural inputs and technologies, extension services and market information. Precisely, cross-sectional household data consisting of two components (crop production component and nutrition and food security component) was collected during a baseline for a project “Increasing Smallholder Farm Productivity, Income and Health through Widespread adoption of Integrated Soil Fertility Management (ISFM) in the Great Lake Regions of Southern Africa”. The data collection involved household survey using a questionnaire. The sampling frames were the smallholder farmers in Goromonzi, Guruve, Hwedza and Mudzi districts. The four districts were selected based on agro-ecological potential and market access. Goromonzi and Guruve districts lie in high potential agro-ecological zones while Mudzi and Hwedza are in low potential zones respectively. Regarding market access, Mudzi has the lowest access compared to the other three districts. Simple random sampling was used to select wards^3^ and individual households from lists provided by resident agricultural extension officers. Crop production and management information were elaborately collected, and included the number of crops grown by the farmers both during the summer and winter seasons, area put under each harvest, inputs used and crop management options adopted e.g. weeding frequency per crop, application of chemicals, and cropping systems used. Information collected on many crops grown, and land area allocation per crop made it easy to calculate the crop diversification index.

### Variable description and statistics

In Table 1 we describe and present the summary statistics of all the variables used in the analysis. Precisely, we report the number of observations, the mean and the standard deviation for each variable within our sample of smallholder farmers.

**Table 1:** Descriptive statistics and variable definitions

	Variable definitions	Count	Mean	SD
Cereal	Cereal output per hectare (kg)	522	1525.739	1388.541
Fcsdaily	Daily food consumption score	594	11.133	5.483
Hfood_security	Binary variable = 1 if household is food secure; 0 otherwise (based of household food insecurity access score)	594	0.221	0.415
Hdds	Household dietary diversity score	594	7.022	2.468
Income	Income from crop sales measured in US$	601	236.952	807.411
Legumeprod	Legume productivity (legume output per hectare measured in kg/ha)	459	1342.412	1995.213
Cd_dum	Binary variable = 1 if smallholder farmer practiced crop diversification; 0 otherwise	575	0.805	0.396
Extension_dum	Binary variable = 1 if farmer received extension services; 0 otherwise	601	0.606	0.489
Info_outputprice	Binary variable = 1 if farmer has information regarding output prices in the market; 0 otherwise	601	0.641	0.480
Roadntwk_good	Binary variable = 1 if the road network is good in the area; 0 otherwise	601	0.290	0.454
Transport_costlow	Binary variable = 1 if transport costs are relatively low in the area; 0 otherwise	601	0.526	0.500
Infoaccess_dum	Binary variable = 1 if farmer has easy access to information; 0 otherwise	599	0.743	0.437
Househ_resp_hhead	Binary variable = 1 if farmer is the household head; 0 otherwise	601	0.567	0.496
Househ_male	Binary variable = 1 if male; 0 otherwise	601	0.757	0.429
Househ_age	Household’s age in years	595	51.447	15.520
Househ_age2	Household’s age squared	595	2887.269	1682.320
Educ_secondary	Binary variable = 1 if farmer completed secondary education or more; 0 otherwise	601	0.478	0.500
Househ_married	Binary variable = 1 if farmer is married; 0 otherwise	601	0.744	0.437
Househ_num_workers	Number of household workers	599	3.249	1.809
Househ_landsize	Size of arable land in hectares	601	2.344	2.661
Househ_landsq	Size of arable land squared	601	12.565	74.541
Distance_market	Distance to the nearest main crop market in kilometers	571	98.732	82.921
Farm_experience	Number of years in farming	595	20.011	14.358
Farm_exp2	Square of number of years in farming	595	606.229	749.987
Occupation_farmer	Binary variable = 1 if full-time farmer; 0 otherwise	601	0.864	0.344
Asset_quintile1	Binary variable = 1 if farmer is in asset quintile 1 (poorest); 0 otherwise	601	0.201	0.401
Asset_quintile2	Binary variable = 1 if farmer is in asset quintile 2; 0 otherwise	601	0.200	0.400
Asset_quintile3	Binary variable = 1 if farmer is in asset quintile 3; 0 otherwise	601	0.200	0.400
Asset_quintile4	Binary variable = 1 if farmer is in asset quintile 4; 0 otherwise	601	0.200	0.400
Asset_quintile5	Binary variable = 1 if farmer is in asset quintile 5 (richest); 0 otherwise	601	0.200	0.400
Wedza	Binary variable = 1 if farmer lives in Wedza district; 0 otherwise	601	0.198	0.399
Mudzi	Binary variable = 1 if farmer lives in Mudzi district; 0 otherwise	601	0.200	0.400
Guruve	Binary variable = 1 if farmer lives in Guruve district; 0 otherwise	601	0.311	0.463
Goromonzi	Binary variable = 1 if farmers lives in Goromonzi district; 0 otherwise	601	0.291	0.455

### Productivity and income

Legume and cereal output per hectare were the two variables used to measure smallholder farm productivity. The average cereal productivity was found to be 1.5 tons per hectare while mean legume productivity was 1.3 tons per hectare. Shown in Table 1 for legume and cereal productivity are logarithmic mean scores for the respective variables. We used income from crop sales as our measure of revenue and shown in Table 1 is the average score of log earnings.

### Food security

We used three food security indicator variables (food consumption score, household food insecurity access score and household dietary diversity score). Food consumption score (FCS) is a weighted score based on dietary diversity, food frequency and nutritional importance of food groups consumed by a household. In this study, we constructed the weighted food consumption score following WFP (2009). The daily score ranges between 0 (food insecure) and 16 (food secure). Mean daily food consumption score in the sample was 11 but reported in Table 1 is the average logarithmic score of the actual food consumption scores.

The extent of household food insecurity is measured by the household food insecurity access score (HFIAS). This score measures the level of food insecurity within the household. We followed Swindale and Bilinsky (2005) and Mango et al. (2014) to compute this score. The household food insecurity score ranges between 0 (food secure) and 27 (food insecure). We then created a dummy variable of food security from the HFIAS score. Mean proportion of food secure households in the sample based on the created dummy for food security is 22 %.

Also, the household dietary diversity score (HDDS) was also used. HDDS is a proxy for nutrient adequacy in a household’s diet. We also follow the same procedures in Swindale and Bilinsky (2005) and Mango et al. (2014) in the computation of this score. The score ranges between 1 (poorly diversified diet) and 14 (highly diversified diet). The mean HDDS score in the sample was 7.

### Crop diversification

**Fig. 1:**
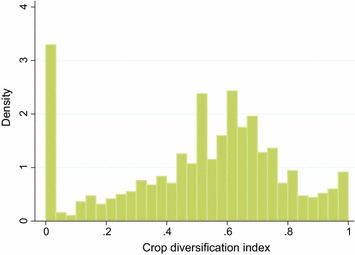
Distribution of the crop diversification index. It shows the distribution of the crop diversification index within our sample of smallholder farmers

From our sample, 81 % of farmers were practicing crop diversification. The Intensity of diversification within the group of diversifiers was high as most of the farmers had an index score >0.5 (Fig. 1).

### Other covariates

Access to extension services within the sample was approximately 61 % while access to crop output market information was at 64 %. Most of the farmers reported their local road networks to be poor as only 29 % of the sample reported to have an excellent road network condition in their area. About 53 % of the farmers reported having relatively low transportation costs in their area. Besides, a larger proportion of the sample (74 %) reported easy access to overall production and marketing information. The majority of the respondents (67 %) were head of households. Our sample was dominated by male headed households (76 %) with a mean age of 51.4 years and a 74 % marriage rate. Regarding education, almost 48 % of the family heads indicated to have attained at least some secondary education at the time of the study. Also, mean arable land size holding within the sample was found to be 2.3 ha and mean size of household labor was 3.2 persons. Mean distance to the nearest main market was 99 km. The farming experience was high within the sample with an average of 20 years. About 86 % of the farmers indicated farming as their main occupation at the time of the survey. We created an asset index with five categories ranked from 1 (poorest) to 5 (richest) via principal components analysis (PCA) (Filmer and Pritchett 2001). Additionally, our sample is made up of; 20 % farmers from Mudzi, 31 % farmers from Guruve, 20 % from Wedza and 29 % from Goromonzi.

## Results and discussion

The OLS estimation results of Eq. (1) for productivity and income and probit regression results for food security and crop diversification [Eq. (2)] are presented in Table 2. In the baseline specifications of Eq. (1) for productivity, income and food security, we do not account for the potential endogeneity of crop diversification in Eq. (1). In other words, we make the assumption that crop diversification is exogenous in Eq. (1). The results for the joint model that controls for the endogeneity bias arising due to the voluntary nature of crop diversification are presented in Table 3. Here, we control for potential endogeneity bias of crop diversification in the outcome equations for crop productivity, income, and food security.

**Table 2:** Impact of crop diversification: baseline specifications

Variables	Log income	Log_fcsdaily	Log_legumeprod	Log_cereal	Hfood security	hdds	Cd_dum
Head of household	−0.078	−0.015	−0.180	−0.049	0.023	−0.200	−0.271*
	(0.202)	(0.027)	(0.218)	(0.131)	(0.053)	(0.141)	(0.151)
Househ_male	−0.035	−0.122	0.824	0.198	0.093	0.150	0.027
	(0.533)	(0.094)	(0.561)	(0.143)	(0.086)	(0.085)	(0.280)
Househ_age	0.017	−0.007	−0.013	0.013	−0.013	−0.075	0.037
	(0.025)	(0.010)	(0.043)	(0.010)	(0.012)	(0.034)	(0.029)
Househ_age2	−0.000	0.000	0.000	−0.000	0.000	0.001*	−0.000
	(0.000)	(0.000)	(0.000)	(0.000)	(0.000)	(0.000)	(0.000)
Educ_secondary	0.528**	−0.083	0.366	0.062	−0.080**	−0.072	0.087
	(0.111)	(0.083)	(0.281)	(0.116)	(0.019)	(0.149)	(0.171)
Househ_married	0.409	0.103	−0.516	0.051	−0.080	−0.550**	−0.290
	(0.757)	(0.090)	(0.491)	(0.112)	(0.093)	(0.152)	(0.286)
Househ_num_workers	0.103	−0.025	−0.066	−0.026	−0.008	0.113	−0.010
	(0.045)	(0.016)	(0.068)	(0.015)	(0.007)	(0.048)	(0.038)
Househ_landsize	0.524***	0.014	0.055	0.022	0.000	0.237	0.158**
	(0.087)	(0.012)	(0.034)	(0.041)	(0.010)	(0.152)	(0.071)
Househ_landsq	−0.014*	0.000	−0.001	−0.001	−0.000	−0.008	−0.005**
	(0.005)	(0.000)	(0.001)	(0.001)	(0.000)	(0.004)	(0.002)
Distance_market	0.001	−0.001	0.000	0.001	0.000	0.000	−0.001
	(0.001)	(0.000)	(0.001)	(0.001)	(0.000)	(0.001)	(0.001)
Farm_experience	0.047	0.001	0.055	−0.014	−0.000	−0.027	−0.055***
	(0.027)	(0.005)	(0.041)	(0.012)	(0.003)	(0.036)	(0.021)
Farm_exp2	−0.001	0.000	−0.001	0.000	0.000	0.001	0.001***
	(0.001)	(0.000)	(0.001)	(0.000)	(0.000)	(0.001)	(0.000)
Occupation_farmer	0.624	0.073	−0.199	0.105	−0.047	−0.312	−0.125
	(0.491)	(0.102)	(0.162)	(0.083)	(0.075)	(0.280)	(0.206)
Asset_quintile2	0.380	−0.036	−0.638*	0.131	−0.025	−0.188	0.642***
	(0.244)	(0.056)	(0.269)	(0.058)	(0.037)	(0.444)	(0.209)
Asset_quintile3	0.404	−0.043	−0.474***	−0.164	−0.054	−0.597	0.326
	(0.305)	(0.059)	(0.074)	(0.133)	(0.064)	(0.406)	(0.210)
Asset_quintile4	0.462	−0.015	−0.897**	0.099	−0.040	−0.201	0.529**
	(0.586)	(0.075)	(0.172)	(0.233)	(0.065)	(0.563)	(0.225)
Asset_quintile5	1.017**	−0.061	−0.432	0.417***	0.000	−0.397	0.589**
	(0.298)	(0.080)	(0.323)	(0.067)	(0.066)	(0.590)	(0.241)
Goromonzi	−0.857***	0.074**	0.472*	0.203*	0.064**	0.092	0.042
	(0.111)	(0.021)	(0.156)	(0.074)	(0.020)	(0.225)	(0.192)
Wedza	−1.783***	0.298***	−1.826***	−1.187***	0.098**	1.648***	0.265
	(0.031)	(0.025)	(0.064)	(0.041)	(0.019)	(0.077)	(0.224)
Mudzi	−2.095***	0.249***	−0.193**	−1.461***	0.038	0.919**	0.970***
	(0.061)	(0.022)	(0.049)	(0.071)	(0.030)	(0.165)	(0.236)
Extension_dum							0.384***
							(0.137)
Info_outputprice							0.370**
							(0.154)
Roadntwk_good							0.184
							(0.160)
Transport_costlow							0.258*
							(0.145)
Infoaccess_dum							−0.297*
							(0.175)
Crop diversification	0.583	−0.095	0.496*	0.192	0.054	0.340**	
	(0.329)	(0.057)	(0.181)	(0.119)	(0.037)	(0.086)	
Observations	538	531	405	484	531	531	538

*** Significant at 1 % level; ** significant at 5 % level; * significant at 10 % level. In parentheses are robust standard errors that account for clustering at the district level. Except for the household food security which binary (hence probit regression estimated), all the other models are based on a continuous outcome variable (hence OLS repression). In the baseline specifications, crop diversification is assumed to be exogenous

**Table 3:** The effect of crop diversification in Zimbabwe: joint estimation results

	Log_income		Log_fcsdaily		Log_legumeprod		Log_cereal		Hfood_security		Hdds	
	Coefficient	SD	Coefficient	SD	Coefficient	SD	Coefficient	SD	Coefficient	SD	Coefficient	SD
Crop diversification	3.498***	(0.648)	0.637***	(0.139)	0.223	(0.526)	1.181***	(0.322)	0.568	(0.571)	3.545***	(0.563)
Head of household	0.108	(0.252)	0.028	(0.056)	−0.189	(0.208)	0.016	(0.086)	0.107	(0.142)	0.033	(0.236)
Gender (male=1)	−0.046	(0.518)	−0.128	(0.096)	0.826	(0.550)	0.194	(0.143)	0.323	(0.236)	0.148	(0.379)
Age	−0.007	(0.052)	−0.013	(0.011)	−0.012	(0.044)	0.004	(0.018)	−0.046	(0.027)	−0.105*	(0.051)
Age squared	0.000	(0.000)	0.000	(0.000)	0.000	(0.000)	−0.000	(0.000)	0.000	(0.000)	0.001*	(0.000)
Secondary education	0.463	(0.276)	−0.099	(0.054)	0.375	(0.216)	0.041	(0.095)	−0.289	(0.152)	−0.138	(0.277)
Married	0.572	(0.520)	0.141	(0.094)	−0.519	(0.532)	0.112	(0.145)	−0.242	(0.238)	−0.331	(0.382)
Number of workers	0.099	(0.064)	−0.027	(0.020)	−0.066	(0.045)	−0.027	(0.026)	−0.029	(0.040)	0.112	(0.065)
Land size	0.418***	(0.099)	0.012	(0.006)	0.038	(0.022)	−0.013	(0.014)	−0.026	(0.026)	−0.001	(0.043)
Land size squared	−0.010*	(0.005)										
Distance to market	0.001	(0.001)	−0.000	(0.000)	0.000	(0.001)	0.002*	(0.001)	0.001	(0.001)	0.000	(0.002)
Farming experience	0.074*	(0.036)	0.006	(0.006)	0.055*	(0.025)	−0.004	(0.013)	0.004	(0.019)	0.011	(0.030)
Farming experience squared	−0.001*	(0.001)	−0.000	(0.000)	−0.001	(0.000)	0.000	(0.000)	−0.000	(0.000)	−0.000	(0.001)
Farmer	0.654	(0.373)	0.076	(0.082)	−0.202	(0.308)	0.112	(0.151)	−0.152	(0.188)	−0.255	(0.325)
Household wealth												
Quintile 2	−0.187	(0.383)	−0.185*	(0.079)	−0.594	(0.303)	−0.073	(0.146)	−0.162	(0.236)	−0.779*	(0.344)
Quintile 3	0.086	(0.382)	−0.134	(0.079)	−0.445	(0.268)	−0.285*	(0.139)	−0.216	(0.219)	−0.898*	(0.375)
Quintile 4	−0.019	(0.424)	−0.150	(0.085)	−0.855**	(0.310)	−0.075	(0.171)	−0.196	(0.240)	−0.672	(0.382)
Quintile 5	0.471	(0.435)	−0.216*	(0.093)	−0.381	(0.280)	0.214	(0.144)	−0.070	(0.259)	−0.910*	(0.409)
Goromonzi district	−0.770*	(0.341)	0.124	(0.064)	0.439	(0.252)	0.236*	(0.104)	0.220	(0.173)	0.057	(0.300)
Wedza district	−1.977***	(0.368)	0.264***	(0.074)	−1.826***	(0.360)	−1.258***	(0.234)	0.283	(0.202)	1.387***	(0.404)
Mudzi district	−2.586***	(0.305)	0.125	(0.068)	−0.153	(0.232)	−1.622***	(0.140)	0.065	(0.228)	0.382	(0.334)
Constant	−1.672	(1.353)	2.226***	(0.317)	6.259***	(1.193)	6.000***	(0.488)	0.173	(0.802)	6.744***	(1.390)
Atanhrho_12	−0.823***	(0.180)	−1.134***	(0.263)	0.092	(0.132)	−0.773*	(0.352)	−0.224	(0.358)	−1.002***	(0.204)
Observations	538		538		538		538		538		538	
Log-likelihood	−1445.7		−558.9		−1018.7		−824.8		−482.1		−1383.2	

*** Significant at 1 % level; ** significant at 5 % level; * significant at 10 % level. Robust standard errors in parentheses. Except for the model for household food security which is estimated via a probit regression model, all the other models are based on a continuous outcome variable and thus use ordinary least squares approach

### Socioeconomic factors influencing crop diversification

The last column in Table 2 presents the probit estimates for the factors associated with the adoption of crop diversification. We found land size holding, farming experience, household wealth, and district of residence, access to agricultural extension services, access to information regarding output prices, low transportation costs and general information access to be significant in explaining variation in crop diversification decision. Precisely, land size holding, asset quintile, district, and access to extension services, access to output prices information and low transportation costs had a positive and significant influence on the decision to diversify crops. On the contrary, farming experience, and access to general information negatively influenced the crop diversification decision.

A one-acre increase (decrease) in land size accessed by the household was found to be associated with 15.8 % increase (decrease) in the probability of adopting crop diversification. Access to land as a resource is a major factor that determines the number of crops that can be grown given a set of resources. This result, suggests that farmers with relatively larger pieces of land are more likely to diversify than their counterparts. Previous studies have found crop diversification to be more feasible on relatively larger farms [see Ashfaq et al. (2008); Weiss and Briglauer (2000)]. Asset quintile, a measure of wealth was also found to be positively related to crop diversification. Compared to households in the lowest asset quintile (asset quintile 1), families in higher wealth groups were found to be positively correlated with the decision to diversify in crop production. Precisely, households in the second, fourth and fifth asset quintile categories were associated with 64.2, 52.9, and 58.9 % more chance of adopting crop diversification than their counterparts in the first quintile category respectively. This finding could be explained by the fact that the decision to diversify need support in terms of access to resources e.g. farming inputs for it to be feasible. Farmers with access to resources can easily adopt crop diversification, unlike their counterparts. The location was also found to positively influence the decision to diversify in crop production. Precisely, farming households in Mudzi district were found to have a 97 % chance of adopting crop diversification than their counterparts in the reference district (Guruve). This result could be related to different agro-ecological factors in different districts analyzed in this study. Mudzi is a low agro-potential and low market access district. Agro potential and market access can influence the farmer’s decision to diversify into crop production. Farmers in low agro-potential and low market access regions like Mudzi can be motivated to try growing different crops for them to guard against production risk and associated marketing challenges. Access to extension advice is important in aiding smallholder farmer production decisions since it can be a reliable source of technical advice on current knowledge, better germplasm and other relevant production information (Mango et al. 2015). Farmers with access to extension services had 38.4 % more chance of adopting a diversified cropping system than their counterparts (those without access to extension). Extension workers have technical knowledge on crop production and management aspects that can assist farmers to implement their crop diversification decisions. Farmers with access to output prices information had a 37.0 % chance of adopting crop diversification than their counterparts. Output prices can motivate farmers to venture or expand production of a certain bundle of crops and hence can influence crop diversification. Also, low transportation costs were also found to have a positive bearing on the decision to diversify. Farmers experiencing low transportation costs had a 25.8 % chance of adopting a diversified cropping system than their counterparts (those experiencing higher transport costs). This finding could be explained by the fact that, low transportation costs lower production and marketing costs, hence farmers being faced by low transaction costs are more likely to diversify when compared to their counterparts.

Farming experience and general access to information were found to negatively influence the decision to diversify into crop production. Precisely, a 1 year increase (decrease) in farming experience was found to be associated with a 5.5 % decrease (increase) in the propensity to adopt crop diversification whilst access to general information was associated with a 29.7 % less chance of adopting a diversified cropping strategy. This implied that, in the study area farmers with more years of farming experience and more access to general information were less likely to venture into crop diversification. This could be explained by the fact that, experience in farming alone cannot be a sufficient condition to diversify, there is need for other supporting factors such as the need to adapt to current conditions e.g. climate (which may be different from the past) and availability of resources to support diversification action. Access to general information can be meaningless to farming decisions; there is a need for specific information on certain practices for a farmer to carefully consider adopting or dis-adoption.

### Impact of crop diversification on productivity, income and food security

Table 2 also present the estimation results for our baseline specification, i.e. Equation (1) estimated without controlling for selection bias. In this case, we estimated OLS models for productivity and income outcomes and a probit regression model for food security. The OLS and probit regression estimates show significant associations between crop diversification and legume productivity and household dietary diversity. We fail to find any statistically significant associations between crop diversification and income, daily consumption, cereal productivity and food security. Various other socioeconomic variables are shown to have a significant influence on our outcome variables; log_income (education, land size, asset quintile and district), log_fcsdaily (district), Log_legumeprod (asset quintile and district), Log_cereal (asset quintile and district), Hfood security (secondary education and district) and hdds (age, marital status and district). For brevity, we do not focus much on interpreting the coefficient estimates of other factors on our outcome variables. We thus focus only on interpreting the results for the observed associations between crop diversification and productivity, income and food security measures.

Table 3 presents the estimation results from joint estimation of Eqs. (1) and (2). As mentioned earlier, joint estimation of the system of equations allows us to control for endogeneity bias created by the selectivity bias of crop diversification in the equations productivity, income and food security. The primary measure of selection bias is the reported *atanhrho* at the bottom of Table 3. The *atanhrho* values reported here are the arc-hyperbolic tangents of the rhos ($\rho$) to make them unbounded. A positive value of *atanhrho* indicates that, there are some unobserved factors that positively impact crop diversification and the main outcome variable. In other words, there is evidence of self-selection in the practice of crop diversification by smallholder farmers in Zimbabwe. In particular, we observe that, the *atanhrho_12* was significant in four out of the six outcome equations; log_income, log_fcsdaily, log_cereal and hdds. The positive values of *atanhrho_12* indicate that there are some omitted variables affecting both the outcome variables and crop diversification positively. The reverse can also be said about the observed negative signs of the *atanhrho_12*.

The general observation from the joint estimation results in Table 3 indicate that crop diversification has a positive and significant impact on cereal crop productivity, crop income, and two food security indicators (food consumption score (FCS) and household dietary diversity score (HDDS). Another interesting finding from our findings is that, failure to control for self-selection in crop diversification results in an under-estimation of the true impact of crop diversification on the outcomes. For example, crop diversification has a positive and statistically insignificant impact on income with a coefficient of 0.583. However, after controlling for selection bias, the impact becomes positive and statistically significant with a coefficient of 3.495. Similar findings can also be observed for crop diversification’s impact on log_fcsdaily, log_cereal and hdds. This highlights the importance of a model that controls for selection bias, another novel contribution of this study.

In order to check the robustness of our findings we also estimated an instrumental variables regression to examine the effect of crop diversification on selected productivity and food security outcomes and income. The 4 presents the estimation results from the instrumental variables (IV) regression. For brevity, we only considered the impact of crop diversification on cereal productivity, income and daily food consumption score. We instrument crop diversification by the binary variable equals 1 if a smallholder farmer had at least one contact with an agricultural and extension services officer and 0 otherwise. In Table 4, we show the regression coefficient estimate and its significance as well as the F-test for the joint significance of the instrument in the first stage regression model. As argued by Staiger and Stock (1997), the estimators may be biased by the presents of weak instruments if the observed F-test for joint significance does not exceed 10. We raise caution on interpreting the statistically positive and significant impact of crop diversification on cereal productivity since this might be affected by the problem of weak instruments as evidenced by an F-test for joint significance of the instrument of 7.947. However, for crop income and daily consumption score, we find F-tests of larger than 10 and a positive and significant impact of crop diversification on crop income only. We fail to find a significant impact of crop diversification on daily consumption. The fact that the IV results are in agreement with the results from the joint model makes our results more robust to different model specifications.

**Table 4:** Effect of crop diversification on productivity, income and food security in Zimbabwe

Variables	(1)	(2)	(3)
	Log_cereal	Log_income	Log_fcsdaily
Extension services contact	2.253**	11.413***	0.543
	(1.051)	(3.743)	(0.397)
Observations	484	538	531
First stage F-statistic	7.947	10.96	11.42

*** Significant at 1 % level; ** significant at 5 % level; * significant at 10 % level. In parentheses are robust standard errors. Crop diversification is instrumented by a dummy variable = 1 if farmer had any contact with agricultural extension workers; 0 otherwise. In all the specifications we include controls for district fixed effects, farmer’s age, experience, gender, education, and other dummy variables for other employment, household wealth and marital status

Moreover, the positive and significant impact we find of crop diversification on productivity, crop income, nutrition and food security is also in line with related studies that have investigated the impact of other climate-smart technology strategies other than crop diversification on productivity and crop income using almost similar identifying restrictions. For example, Manda et al. (2016) investigated the impact of sustainable agricultural practices on maize yields and incomes of smallholder farmers in Zambia. Using a binary variable for contact with extension officers as an exclusion restriction, they find a positive and significant impact of sustainable agriculture practices such as maize and legume rotation and residue retention on productivity and incomes of Zambian smallholder farmers.

### Crop diversification and productivity

Our results report positive and significant impacts of crop diversification on cereal crop productivity. This implies that diversified cropping systems including cereal and legume intercrops can improve productivity of the principal crop (which is usually maize in Zimbabwe). This could be as a result of the improved soil fertility in legume and cereal mixtures. Smith and Read (2008) found diversity through crop rotations of greater cover crops and nitrogen fixing crops to increase the yield of the primary crop. The main probable options for improvement in crop productivity with diversification is that, crop mixtures are more likely to be effective in suppressing diseases and pests, increasing soil fertility and improving the efficiency of local agro ecological systems. In literature, diversified cropping systems have been found to work in raising productivity by increasing natural enemies of insects and pests, breaking disease cycles (Larkin et al. 2010; Ojaghian et al. 2012), suppressing weeds and volunteer crop plants (Campiglia et al. 2010), modifying the microenvironment within the crop canopy and or making pest and disease penetration more difficult (Krupinsky et al. 2002; Lin 2011). This makes crop diversification very important to smallholder farmers in adapting to climate change and variability as a number of challenges problematic in smallholder farming systems can be reduced in severity hence helping in building long term resilience to climate variability and change.

### Crop diversification and income

It also evident in our findings that crop diversification significantly improves income from agricultural activities. Increased production from diversified cropping systems and increased production stability are the probable explanations for improved income as a result of crop diversification. This is an important finding considering that climate change and variability reduces crop yields and increases susceptibility to total crop failure. Adopting a more diversified cropping system is therefore an important adaptation option as it reduces production risks hence bringing improved production stability.

### Crop diversification and food security

In addition, crop diversification was found to have a positive and statistically significant impact on food security and nutrition indicators (food consumption score and household dietary diversity). This implies that besides improving productivity, increasing production and income stability, crop diversification also has a direct effect on food availability and nutrition. This is mainly because crop diversification will improve yields, bring crop yield stability and also that crop insurance effect (Mugendi Njeru 2013; Yachi and Loreau 1999), since if one crop fails the farmer can depend on the other crop. This will have a direct impact on food security and nutrition in smallholder farming systems since traditionally the main aim will be to sustain the family and selling surplus where possible. This makes crop diversification a more important climate smart option as improving food security and diet options will help in building resilience to intensifying climate change and variability effects by smallholder farmers. According to Mugendi Njeru (2013) crop diversification not only allows more efficient utilization of agro ecological processes, but also provides diversity for human diet and improve income which improves the purchasing power for the household for buying other foods.

## Conclusion and recommendation

In this study, we examined the effects of crop diversification on productivity, income, food security and nutrition in Zimbabwe’s smallholder farming community. Our results show a strong and positive association between crop diversification and crop productivity, crop income, food security and nutrition measures. The merits of crop diversification that explain the positive association with crop productivity, crop income, food security and nutrition include its ability to; improve soil fertility, suppress diseases and pests (by increasing natural enemies of pests and breaking disease cycles), suppress weeds and volunteer crops, improve efficiency of agro-ecological systems which in turn reduces crop production risks, improves production stability, yields, crop income and diversity of human diet. We conclude that, with clear evidence of growing stress in smallholder farming systems from climate change and variability in southern Africa, greater implementation of diversified cropping systems especially those currently less diversified, can significantly improve yields, income, food security and nutrition. This is mainly because of the advantages that come with diversified cropping systems which largely reduce risks of crop production, gives more income options to the farmer, and makes production on the farm more stable. This makes crop diversification an equally climate smart agriculture option for smallholder farmers that can go a long way in providing the necessary ammunition to adapt to intensifying climate change effects in Zimbabwe and other similar smallholder farming systems in the region.

## Footnotes

1 We estimated crop diversification as an index of concentration that ranges between 0 (specialization) and 1 (complete diversification). Any value above zero signifies diversification. Precisely, we estimated the index by subtracting the Herfindahl index (HI) from one (1-HI), where HI was calculated as follows: ${\text{HI}} = \sum\nolimits_{i = 1}^{n} {s_{i}^{2} }$ , where $S_{i} = \frac{{A_{i} }}{{\sum\nolimits_{i = 1}^{n} {A_{i} } }}$ and S_i_ =  proportion of ith crop, A_i_ = Area under ith crop, $\sum\nolimits_{i = 1}^{n} {A_{i} }$ = Total cropped area, and *i* = 1, 2, 3, 4,…, n (number of crops considered). In this study we used eight crops common in the study areas to calculate the index.

2 We combined information on ownership of land and non-land assets, livestock ownership and household dwelling characteristics to create a living standards measure, the asset index, using principal components analysis (PCA) (Filmer and Pritchett 2001; Rutstein and Johnson 2004). The first principal component is then used as an overall measure of living standards. For a detailed and more technical exposition, see McKenzie (2005).

3 A ward in Zimbabwe is the second smallest administrative unit after the village and consist of several villages.
